# Predictors of eHealth Usage: Insights on The Digital Divide From the Health Information National Trends Survey 2012

**DOI:** 10.2196/jmir.3117

**Published:** 2014-07-16

**Authors:** Emily Kontos, Kelly D Blake, Wen-Ying Sylvia Chou, Abby Prestin

**Affiliations:** ^1^Harvard School of Public HealthDepartment of Social and Behavioral SciencesBoston, MAUnited States; ^2^National Cancer InstituteDivision of Cancer Control and Population SciencesBethesda, MDUnited States; ^3^United States Food and Drug AdministrationCenter for Tobacco Products, Office of ScienceSilver Spring, MDUnited States

**Keywords:** health communication, communication barriers, Internet, consumer health information

## Abstract

**Background:**

Recent eHealth developments have elevated the importance of assessing the extent to which technology has empowered patients and improved health, particularly among the most vulnerable populations. With noted disparities across racial and social groups in chronic health outcomes, such as cancer, obesity, and diabetes, it is essential that researchers examine any differences in the implementation, uptake, and impact of eHealth strategies across groups that bear a disproportionate burden of disease.

**Objective:**

The goal was to examine eHealth use by sociodemographic factors, such as race/ethnicity, socioeconomic status (SES), age, and sex.

**Methods:**

We drew data from National Cancer Institute’s 2012 Health Information National Trends Survey (HINTS) (N=3959) which is publicly available online. We estimated multivariable logistic regression models to assess sociodemographic predictors of eHealth use among adult Internet users (N=2358) across 3 health communication domains (health care, health information–seeking, and user-generated content/sharing).

**Results:**

Among online adults, we saw no evidence of a digital use divide by race/ethnicity. However, there were significant differences in use by SES, particularly for health care and health information–seeking items. Patients with lower levels of education had significantly lower odds of going online to look for a health care provider (high school or less: OR 0.50, 95% CI 0.33-0.76) using email or the Internet to communicate with a doctor (high school or less: OR 0.46, 95% CI 0.29-0.72), tracking their personal health information online (high school or less: OR 0.53, 95% CI 0.32-0.84), using a website to help track diet, weight, and physical activity (high school or less: OR 0.64, 95% CI 0.42-0.98; some college: OR 0.67, 95% CI 0.49-0.93), or downloading health information to a mobile device (some college: OR 0.54, 95% CI 0.33-0.89). Being female was a consistent predictor of eHealth use across health care and user-generated content/sharing domains, whereas age was primarily influential for health information–seeking.

**Conclusions:**

This study illustrates that lower SES, older, and male online US adults were less likely to engage in a number of eHealth activities compared to their counterparts. Future studies should assess issues of health literacy and eHealth literacy and their influence on eHealth engagement across social groups. Clinical care and public health communication efforts attempting to leverage Web 2.0 and 3.0 platforms should acknowledge differential eHealth usage to better address communication inequalities and persistent disparities in health.

## Introduction

The movement in the past decade toward patient-centered care has increasingly emphasized patient empowerment in health care. In particular, the Chronic Care Model, characterized by the interaction between an “informed and activated patient” and a “prepared and proactive practice team” has been highlighted as a fundamental model for optimum care [1,2]. Alongside the changing tide in health care delivery, there has been a revolution in information technology. With the development of new technology and Web 2.0 and 3.0 communication media, the field of eHealth has emerged and with it a plethora of new opportunities for individuals to access and exchange health information, manage their health through electronic platforms, and participate in “peer-to-peer health care” [3-5]. These online opportunities have been identified as a means to better enable patient empowerment and self-management of care [6].

The field of eHealth has enabled public health and medical practitioners to communicate with patients in both traditional and novel ways to address health concerns such as diabetes management [7], heart health [8], cancer prevention and health promotion activities [9-11], and smoking cessation [12,13]. Online strategies range from adaptations of more traditional communication methods, such as the delivery of tailored information and the creation of support networks [14], to more innovative developments, such as the implementation of smartphone applications for disease prevention and management [15-17].

These eHealth developments have elevated the importance of assessing the extent to which technology has empowered patients and improved health in general and among the most vulnerable populations in particular [18-21]. With noted disparities across racial and social groups in chronic health outcomes, such as cancer, obesity, and diabetes, it is essential that researchers thoughtfully examine any differences in the implementation, uptake, and impact of eHealth strategies across groups that bear a disproportionate burden of disease [20,22,23]. Current eHealth studies are limited in that many, such as those published by the Pew Internet & American Life Project, report national percentages without rigorous statistical control to determine what factors may be true drivers of any eHealth disparities. Other research has focused on issues of access to technology based on the original concept of the digital divide, which formulated 2 groups—those with access to the Internet and those without [5,24]. Gaps in access to the Internet have been persistent in that lower socioeconomic status, minority racial/ethnic groups, older age, and poorer health, among others, are associated with decreased access to the Internet [19,21,25-29]. These access patterns can lead to differential access to health information that might intensify health disparities [30]. Yet, this dichotomous oversimplification wrongly suggests that all “haves” use the Internet in a similar manner.

However, the limited number of published studies focused on use of the Internet for health point to noted communication inequalities or differences in use and engagement across important racial and social groups [18,22,31]. For example, those in the lowest income and education brackets are shown to be considerably less likely to seek out health information online compared to those in higher income and education brackets. Similarly, non-Hispanic blacks and Hispanics are significantly less likely to seek out health information online compared to their non-Hispanic white counterparts, yet these differences by race/ethnicity have begun to narrow overall. Recent unadjusted Pew Internet & American Life Project data highlight differences in topic-specific seeking behavior in that more non-Hispanic blacks and Hispanics report using the Internet to find information on how to lose weight and pregnancy compared to non-Hispanic whites, whereas a larger percentage of non-Hispanic whites report using the Internet to find information on a specific disease or problem [5,20,32,33]. These differences in eHealth use are of importance to public health practitioners and health care providers in that these communication behaviors could lead to significant health-related disparities [22].

Evidence in support of communication inequalities in health-related Internet use is building [20,34,35]; however, we lack a clear understanding of comprehensive differences by sociodemographic factors, such as race/ethnicity, socioeconomic status (SES), and sex in the utility of the Internet for eHealth tasks. Past studies have primarily examined online disparities in isolation, have not adequately adjusted for confounding factors that could drive use, or have used older datasets that do not reflect the ever-changing digital landscape [5,34,36-38].

This study aims to employ an up-to-date, comprehensive examination of eHealth use by sociodemographic factors to illustrate potential profiles of disparities across a number of communication domains. We hope this work will assist future health communication interventions and efforts that seek to use the Internet, email, and social media to reach and engage underserved populations.

## Methods

Data for this study were drawn from the National Cancer Institute’s 2012 Health Information National Trends Survey (HINTS). HINTS is a nationally representative survey of the US noninstitutionalized adult population that collects data on the American public’s need for, access to, and use of health-related information [39]. HINTS is publicly available online [40]. Data used in this study are from HINTS 4 Cycle 1, collected from October 2011 to February 2012 (N=3959) through mailed questionnaire. The sample design was a 2-stage stratified sample with addresses selected from a comprehensive United States Postal Service national residential file, and individual respondents were selected per each household in the sample. The final response rate for HINTS 2012 was 36.7%. Further details on survey design and sampling strategies are published elsewhere [41].

**Figure 1:**
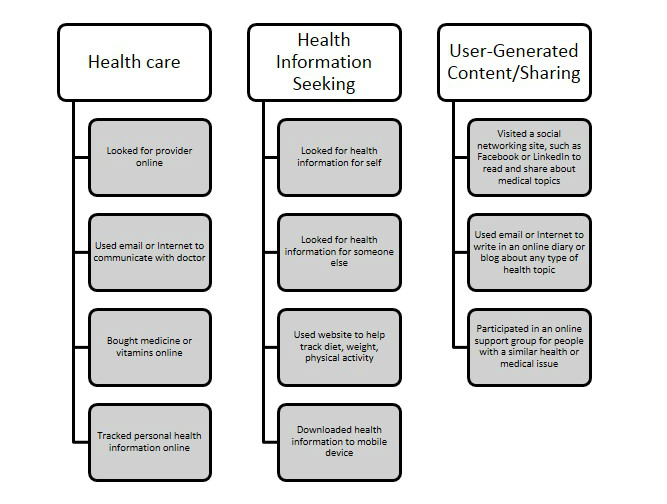
Health communication domains.

To assess hypothesized differences in eHealth usage and engagement, we used 11 HINTS variables that were asked of those respondents who reported yes to ever going online to access the Internet or World Wide Web or to send and receive email (N=2358). The 11 eHealth tasks are presented in 3 domains relevant to health communication (health care, health information-seeking, and user-generated content/sharing). Items were grouped into domains in effort to illustrate trends across eHealth tasks and for purposes of informing future health communication-related interventions [42]. The categorization of items into domains was informed by both mass communication theory, such as uses and gratifications theory, as well as recent health care policies, specifically the Affordable Care Act and Healthy People 2020, in which there is interest to track progress in goal achievement [43-45]. For example, one of the goals outlined in Healthy People 2020 is aimed at improving access to comprehensive, quality health care services, research is emerging that correlates increased engagement with the Internet and access to health care services [46]. The eHealth items assessed in this study were “In the past 12 months, have you used the Internet to look for health or medical information for yourself?” (yes/no) and “In the past 12 months, have you used the Internet for any of the following reasons?” (yes/no) as listed in Figure 1.

For the purpose of this analysis, primary predictor variables included in each model represent sociodemographic characteristics: place of birth, race/ethnicity, home ownership, education, income, age, and sex. Hot-deck imputation was used to replace missing responses with imputed data for the race and ethnicity variables in HINTS 2012. With this approach, the resulting distribution preserved the distribution of values observed for respondents.

All models adjusted for occupational status, marital status, children, health information–seeking (ever sought health information from any source), regular access to a health care provider, insurance status, health status, personal cancer history, and family cancer history.

We used multivariable logistic regression to model the fitted odds that SES (education and income), race/ethnicity, age, and sex independently and differentially predicted eHealth usage in the population of online US adults. We used SAS-callable SUDAAN 10.0.1 to account for the complex sampling design used in HINTS and to incorporate jackknife replicate weights needed to compute accurate standard errors. All analyses were weighted to provide nationally representative estimates. We calculated weighted percentages, odds ratios (OR), and 95% confidence intervals (CI) utilizing complete case analyses with listwise deletion for each model (N=2358).

## Results

### Summary

Weighted and unweighted unadjusted prevalence estimates for each dependent variable are presented in Table 1 (demographics) and Table 2 (eHealth tasks). For health care–related tasks, the prevalence of eHealth usage is generally low, with approximately 18.95% (509/2358) of online US adults reporting ever having engaged in activities such as emailing providers, 19.29% (501/2358) tracking health information online, and 17.67% (459/2358) buying medicine online. Slightly more people have used the Internet to search for a health care provider (38.42%, 861/2358). For health information–seeking tasks, eHealth usage is notably more prevalent. Nearly 80% (79.04%, 1833/2358) of online American adults have used the Internet to look for health information for themselves and 57.04% (1342/2358) have used the Internet to look for health information for someone else. Approximately 42.98% (925/2358) have used the Internet in the past year to help with diet, weight, or physical activity, but far fewer have used it to download health information to a mobile device (11.70%, 261/2358). In terms of engagement with user-generated content, only a small proportion of the population (3.26%-4.63%, 76-110/2358 of online US adults) took advantage of interactive Web features, such as participating in an online support group or health-related blog. Use of social networking sites (SNS) for health is a bit higher, with 16.80% (345/2358) of online Americans saying they have visited sites such as Facebook or LinkedIn to read or share about medical topics.

Pursuant to our central research question on potential communication inequalities in eHealth usage, results for our multivariable logistic regression analyses are presented by the domains of health care, health information–seeking, and user-generated content/sharing. Among online adults, there was little evidence of a digital use divide by race/ethnicity. Generally, non-Hispanic blacks, Hispanics, and people of other races were no more or less likely than non-Hispanic whites to engage in eHealth activities. Being of other race was predictive of almost a 3-fold increase in the odds of downloading health information to a mobile device and twice the odds of using the Internet to look for a health care provider. However, the most significant differences in eHealth use were across SES (either by education, income, or both) and by age and sex. Findings are summarized subsequently.

**Table 1:** Weighted and unweighted unadjusted prevalence estimates for sample demographics, Health Information National Trends Survey (HINTS) 4 Cycle 1, October 2011 to February 2012 (N=2358).

Sociodemographics		n	Unweighted %	Weighted %
**Age group**				
	18-34	440	18.66	35.21
	35-49	680	28.84	30.07
	50-64	824	34.94	24.63
	65-74	292	12.38	7.03
	>75	122	5.17	3.05
**Highest level of school completed**				
	≤High school	401	17.01	23.58
	Some college	761	32.27	35.25
	College graduate or more	1196	50.72	41.17
**Race/ethnicity (imputed)**				
	Non-Hispanic white	1657	70.27	70.43
	Hispanic	215	9.12	11.50
	Non-Hispanic black	322	13.66	10.39
	Non-Hispanic other	164	6.96	7.69
**Annual household income**				
	Less than $20,000	329	13.95	16.79
	$20,000 to <$35,000	313	13.27	14.33
	$35,000 to <$50,000	313	13.27	11.27
	$50,000 to <$75,000	437	18.53	18.69
	>$75,000	821	34.82	33.04
	Missing	145	6.15	5.88
**Sex**				
	Male	931	39.48	48.36
	Female	1427	60.52	51.64
**Ever diagnosed as having cancer?**				
	Yes	311	13.19	7.34
	No	2047	86.81	92.66
**Any family members ever had cancer?**				
	Yes	1506	63.87	62.92
	No/not sure/missing	852	36.13	37.08

**Table 2:** Weighted and unweighted unadjusted prevalence estimates for eHealth tasks, Health Information National Trends Survey (HINTS) 4 Cycle 1, October 2011 to February 2012 (2012) (N=2358).

eHealth Task		n	Unweighted %	Weighted %
**Ever looked for information about health or medical topics from any source?**				
	Yes	2132	90.42	88.79
	No	226	9.58	11.21
**In the past 12 months, used email or Internet to communicate with a doctor or doctor’s office?**				
	No	1849	78.41	81.05
	Yes	509	21.59	18.95
**In the past 12 months, bought medicine or vitamins online?**				
	No	1899	80.53	82.33
	Yes	459	19.47	17.67
**In the past 12 months, used the Internet to look for a health care provider?**				
	No	1497	63.49	61.58
	Yes	861	36.51	38.42
**In the last 12 months, used the Internet to keep track of personal health information**				
	No	1857	78.75	80.71
	Yes	501	21.25	19.29
**In the past 12 months, used a website to help with diet, weight, or physical activity?**				
	No	1433	60.77	57.02
	Yes	925	39.23	42.98
**In the last 12 months, used the Internet to download health-related info to a mobile device**				
	No	2097	88.93	88.30
	Yes	261	11.07	11.70
**In the past 12 months, used the Internet to look for health or medical information for self?**				
	No	525	22.3	21
	Yes	1833	77.7	79
**In the past 12 months, used the Internet to look for health or medical information for someone else?**				
	No	1016	43.09	42.96
	Yes	1342	56.91	57.04
**In the past 12 months, participated in an online support group for people with a similar health or medical issue?**				
	No	2248	95.34	95.37
	Yes	110	4.66	4.63
**In the past 12 months, visited a social networking site to read and share about medical topics?**				
	No	2013	85.37	83.20
	Yes	345	14.63	16.80
**In the past 12 months, wrote in an online diary or blog about any type of health topic?**				
	No	2282	96.78	96.74
	Yes	76	3.22	3.26

### Health Care

Online adults with the lowest levels of education were significantly less likely to use the Internet to look for a health care provider compared to those with a college degree or more (OR 0.50, 95% CI 0.33-0.76) (Table 3). The youngest adults surveyed (18-34 years) had more than twice the odds of engaging in online provider searches compared to the oldest group aged 65 years and older (OR 2.24, 95% CI 1.20-4.16). Whereas, women were more likely than men to search for a health care provider online (OR 1.53, 95% CI 1.14-2.04).

Among online adults, we again saw evidence of a usage gap by education and sex with regard to using email or the Internet to communicate with a doctor or doctor’s office. Those with a high school degree or less were less likely to engage in this activity compared to those with a college degree or more (OR 0.46, 95% CI 0.29-0.72). Additionally, women were more likely than men to have communicated with a provider by email or Internet (OR 1.52, 95% CI 1.06-2.19).

Education and sex were also significant predictors of tracking personal health information online. High school graduates and those with lower levels of education were less likely than college graduates to have tracked this information online (OR 0.53, 95% CI 0.32-0.84), whereas women were 1.5 times as likely to have done so than men were (OR 1.52, 95% CI 1.06-2.19).

We saw different patterns in use when examining who purchased medicine or vitamins online. Income level and place of birth were significant predictors; those earning <$20,000 and between $20,000 and <$35,000 annually were significantly less likely than those in the highest income category to have made medicine or vitamin purchases online (OR 0.34, 95% CI 0.12-0.95 and OR 0.38, 95% CI 0.16-0.90, respectively), whereas those not born in the United States had more than 2.5 times the odds of having done so (OR 2.64, 95% CI 1.37-5.09).

**Table 3:** Multivariable logistic regression models for odds^a^ of reporting yes to eHealth usage by socioeconomic status and race/ethnicity, Health Information National Trends Survey (HINTS), 2012.

Sociodemographics		Looked for health care provider		Used email or Internet to communicate with doctor		Bought medicine or vitamins online		Tracked personal health information online	
		OR	95% CI	OR	95% CI	OR	95% CI	OR	95% CI
**Education**									
	≤High school degree	0.50	0.33, 0.76	0.46	0.29, 0.72	0.67	0.34, 1.32	0.53	0.32, 0.84
	Some college	0.73	0.49, 1.09	0.71	0.50, 1.02	0.99	0.67, 1.46	0.75	0.50, 1.13
	>College degree (ref)	1.00	1.00, 1.00	1.00	1.00, 1.00	1.00	1.00, 1.00	1.00	1.00, 1.00
**Born in United States**									
	No	1.29	0.73, 2.26	1.03	0.49, 2.14	2.64	1.37, 5.09	1.19	0.58, 2.46
	Yes (ref)	1.00	1.00, 1.00	1.00	1.00, 1.00	1.00	1.00, 1.00	1.00	1.00, 1.00
**Race/ethnicity**									
	Hispanic	0.73	0.44, 1.16	1.21	0.69, 2.13	1.01	0.49, 2.10	1.04	0.60, 1.81
	Non-Hispanic black	1.52	0.72, 3.18	0.94	0.49, 1.79	1.39	0.50, 3.91	1.23	0.73, 2.06
	Other race	2.02	1.01, 4.04	1.68	0.91, 3.11	0.79	0.43, 1.46	1.82	0.81, 4.07
	Non-Hispanic white (ref)	1.00	1.00, 1.00	1.00	1.00, 1.00	1.00	1.00, 1.00	1.00	1.00, 1.00
**Home ownership**									
	Rent or occupy without rent	1.06	0.67, 1.68	1.04	0.72, 1.51	0.81	0.51, 1.28	1.19	0.86, 1.64
	Own (ref)	1.00	1.00, 1.00	1.00	1.00, 1.00	1.00	1.00, 1.00	1.00	1.00, 1.00
**Annual household income**									
	<$20,000	0.86	0.38, 1.97	0.37	0.13, 1.02	0.34	0.12, 0.95	0.78	0.31, 1.94
	$20,000 to <$35,000	0.70	0.35, 1.38	0.49	0.23, 1.06	0.38	0.16, 0.90	0.81	0.45, 1.46
	$35,000 to <$50,000	1.51	0.95, 2.40	0.67	0.40, 1.13	0.70	0.32, 1.54	0.64	0.36, 1.15
	$50,000 to <$75,000	1.00	0.64, 1.58	0.71	0.47, 1.06	0.91	0.60, 1.37	1.31	0.80, 2.14
	>$75,000 (ref)	1.00	1.00, 1.00	1.00	1.00, 1.00	1.00	1.00, 1.00	1.00	1.00, 1.00
**Age**									
	18-34	2.24	1.20, 4.16	0.91	0.48, 1.70	0.85	0.46, 1.58	1.24	0.69, 2.22
	35-49	1.55	0.91, 2.63	0.77	0.47, 1.24	0.68	0.40, 1.14	0.90	0.58, 1.39
	50-64	1.50	0.90, 2.51	0.84	0.51, 1.37	0.73	0.46, 1.16	0.91	0.65, 1.28
	>65 (ref)	1.00	1.00, 1.00	1.00	1.00, 1.00	1.00	1.00, 1.00	1.00	1.00, 1.00
**Sex**									
	Female	1.53	1.14, 2.04	1.47	1.03, 2.09	0.98	0.65, 1.47	1.52	1.06, 2.19
	Male (ref)	1.00	1.00, 1.00	1.00	1.00, 1.00	1.00	1.00, 1.00	1.00	1.00, 1.00

^a^All estimates are weighted. All models control for occupational status, marital status, children, health information–seeking (ever sought health information from any source), regular access to a health care provider, insurance status, health status, personal cancer history, and family history of cancer.

### Health Information–Seeking

Age was the sole predictor of whether online adults used the Internet in the past 12 months to search for health information for themselves (Table 4). Adults aged 18-34 years were 3.5 times as likely and adults aged 35-49 years were nearly 2.5 times as likely as those 65 years and older to use the Internet to search for health information (OR 3.51, 95% CI 1.66-7.44 and OR 2.35, 95% CI 1.17-4.72, respectively).

Age and sex were differentially predictive of using the Internet to search for health or medical information for someone else. Those aged 35-49 years were 1.5 times as likely to have used the Internet for this purpose as those 65 years and older (OR 1.52, 95% CI 1.00-2.31). Women were approximately 1.5 times as likely as men to have done so (OR 1.46, 95% CI 1.06-2.01).

Similarly, we identified gaps by age, education, and income in use of websites to help with diet, weight, or physical activity. Overwhelmingly, those in younger age categories were significantly more likely than those aged 65 and older to have used a website for this purpose: age 18-34 (OR 3.37, 95% CI 2.00-5.69), age 35-49 (OR 2.57, 95% CI 1.66, 3.99), and age 50-64 (OR 2.22, 95% 1.43-3.43). Those with a high school degree or less and those with some college were approximately 35% less likely than college graduates to have done so (OR 0.64, 95% CI 0.42-0.98 and OR 0.67, 95% CI 0.49-0.93, respectively). Additionally, those making less than $20,000 per year were nearly 50% less likely than those in the highest income category to have used the Web for this purpose (OR 0.46, 95% CI 0.24-0.86).

In terms of downloading health-related information to a mobile device, such as an MP3 player, cell phone, tablet computer, or electronic book device, we observed a few usage gaps by education level and race/ethnicity. Those with some college were 46% less likely than those with a college degree to have gone online for this purpose (OR 0.54, 95% CI 0.33-0.89) and those in the lowest education bracket were 58% less likely to engage in this eHealth task, but this did not meet statistical significance (OR 0.42, 95% CI 0.17-1.02). Those of other race were nearly 3 times as likely to download health information to a mobile device compared to their non-Hispanic white counterparts (OR 2.78, 95% CI 1.33-5.86).

**Table 4:** Multivariable logistic regression models for odds^a^ of reporting yes to eHealth usage, by socioeconomic status and race/ethnicity, Health Information National Trends Survey (HINTS), 2012.

Sociodemographics		Looked for health information for self		Looked for health information for someone else		Used website to help track diet, weight, physical activity		Downloaded health information to mobile device	
		OR	95% CI	OR	95% CI	OR	95% CI	OR	95% CI
**Education**									
	≤High school degree	0.64	0.41, 1.01	0.72	0.46, 1.10	0.64	0.42, 0.98	0.42	0.17, 1.02
	Some college	0.67	0.44, 1.02	0.70	0.46, 1.05	0.67	0.49, 0.93	0.54	0.33, 0.89
	>College degree (ref)	1.00	1.00, 1.00	1.00	1.00, 1.00	1.00	1.00, 1.00	1.00	1.00, 1.00
**Born in United States**									
	No	1.43	0.68, 2.99	1.29	0.67, 2.48	1.39	0.77, 2.49	1.36	0.65, 2.88
	Yes (ref)	1.00	1.00, 1.00	1.00	1.00, 1.00	1.00	1.00, 1.00	1.00	1.00, 1.00
**Race/ethnicity**									
	Hispanic	0.62	0.27, 1.45	0.89	0.51, 1.57	1.58	0.80, 3.11	1.47	0.57, 3.82
	Non-Hispanic black	1.21	0.66, 2.23	1.32	0.65, 2.68	1.00	0.55, 1.82	1.73	0.88, 3.42
	Other race	1.13	0.40, 3.20	1.78	0.94, 3.34	1.40	0.75, 2.60	2.78	1.33, 5.86
	Non-Hispanic white (ref)	1.00	1.00, 1.00	1.00	1.00, 1.00	1.00	1.00, 1.00	1.00	1.00, 1.00
**Home ownership**									
	Rent or occupy without rent	0.89	0.59, 1.33	1.13	0.72, 1.79	1.06	0.72, 1.57	1.18	0.62, 2.24
	Own (ref)	1.00	1.00, 1.00	1.00	1.00, 1.00	1.00	1.00, 1.00	1.00	1.00, 1.00
**Annual household income**									
	<$20,000	0.84	0.39, 1.80	1.06	0.52, 2.15	0.46	0.24, 0.86	1.28	0.59, 2.80
	$20,000 to <$35,000	0.83	0.40, 1.74	1.45	0.72, 2.90	0.72	0.39, 1.32	0.88	0.37, 2.13
	$35,000 to <$50,000	0.94	0.50, 1.75	1.10	0.60, 2.00	0.59	0.34, 1.05	0.81	0.38, 1.75
	$50,000 to <$75,000	0.87	0.47, 1.59	1.02	0.68, 1.52	1.12	0.74, 1.72	1.09	0.61, 1.96
	>$75,000 (ref)	1.00	1.00, 1.00	1.00	1.00, 1.00	1.00	1.00, 1.00	1.00	1.00, 1.00
**Age**									
	18-34	3.51	1.66, 7.44	1.31	0.74, 2.31	3.37	2.00, 5.69	1.78	0.77, 4.09
	35-49	2.35	1.17, 4.72	1.52	1.00, 2.31	2.57	1.66, 3.99	0.89	0.43, 1.84
	50-64	1.62	0.87, 3.02	1.44	0.95, 2.19	2.22	1.43, 3.43	1.32	0.66, 2.62
	>65 (ref)	1.00	1.00, 1.00	1.00	1.00, 1.00	1.00	1.00, 1.00	1.00	1.00, 1.00
**Gender**									
	Female	1.43	0.96, 2.12	1.46	1.06, 2.01	1.25	0.93, 1.70	1.21	0.75, 1.93
	Male (ref)	1.00	1.00, 1.00	1.00	1.00, 1.00	1.00	1.00, 1.00	1.00	1.00, 1.00

^a^All estimates are weighted. All models control for occupational status, marital status, children, health information–seeking (ever sought health information from any source), regular access to a health care provider, insurance status, health status, personal cancer history, and family history of cancer.

### Engagement in User-Generated Content and Social Media

We saw differences in health-related social media use by SES, sex, and age among online adults (Table 5). Both lower education and lower income were predictive of using SNS, such as Facebook, to read or share about medical topics. Those with some college were more than 1.5 times as likely as those with a college degree to engage in this eHealth activity (OR 1.59, 95% CI 1.06-2.39). Similarly, those with household incomes less than $20,000 were more than twice as likely to use SNS to read or share about health (OR 2.12, 95% CI 1.04-4.29) and those earning $20,000-$35,000 were also nearly twice as likely to engage in this activity compared to those with a household income of $75,000 or more, although this did not meet statistical significance (OR 1.82, 95% CI 0.99-3.32). Moreover, we saw a fine gradation of effect from the youngest to oldest age categories for SNS health use. Compared to those aged 65 and older, those aged 18-34 were nearly 3 times more likely to have gone online for this purpose and those aged 35-49 were more than twice as likely to have done so (OR 2.81, 95% CI 1.13-7.00 and OR 2.27, 95% CI 1.00-5.17, respectively).

Participation in an online support group for people with a similar medical issue was more prominent among women; women were nearly 3 times as likely to participate in an online support group compared to men (OR 2.79, 95% CI 1.20-6.51). Those with a household income of $50,000-$75,000 were more than twice as likely compared to the highest income bracket to do so (OR 2.22, 95% CI 1.04-4.73). In terms of writing an online blog about a health topic, women were more than 4 times as likely as men to have done so (OR 4.31, 95% CI 1.78-10.42).

Having a connection to cancer was also predictive of engaging in user-generated content for health. Respondents with a family history of cancer were nearly 3 times as likely to have participated in an online support group than those without a family cancer experience (OR 2.96, 95% CI 1.00-3.83). Cancer survivors were nearly 3 times as likely to have written in a blog about a health topic compared to those with no cancer history (OR 2.93, 95% CI 1.00-8.63).

**Table 5:** Multivariable logistic regression models for odds^a^ of reporting yes to eHealth usage, by SES and race/ethnicity, Health Information National Trends Survey (HINTS), 2012.

Sociodemographics		Visited a social networking site to read and share about medical topics		Used email or Internet to write in an online diary or blog about any type of health topic		Participated in an online support group for people with a similar health or medical issue^b^	
		OR	95% CI	OR	95% CI	OR	95% CI
**Education**							
	≤High school	1.11	0.64, 1.92	1.05	0.38, 2.90	0.90	0.27, 3.00
	Some college	1.59	1.06, 2.39	1.13	0.45, 2.85	1.44	0.79, 2.64
	College degree or more (ref)	1.00	1.00, 1.00	1.00	1.00, 1.00	1.00	1.00, 1.00
**Born in United States**							
	No	0.77	0.42, 1.39	1.43	0.31, 6.68	1.96	0.36, 8.86
	Yes (ref)	1.00	1.00, 1.00	1.00	1.00, 1.00	1.00	1.00, 1.00
**Race/ethnicity**							
	Hispanic	0.90	0.39, 2.09	0.62	0.12, 3.20	1.91	0.61, 5.95
	Non-Hispanic black	1.24	0.57, 2.67	1.32	0.39, 4.53	0.57	0.17, 1.88
	Other race	1.08	0.48, 2.43	1.33	0.26, 6.83	1.80	0.50, 6.45
	Non-Hispanic white (ref)	1.00	1.00, 1.00	1.00	1.00, 1.00	1.00	1.00, 1.00
**Home ownership**							
	Rent or occupy without rent	1.23	0.72, 2.10	1.18	0.37, 3.71	0.83	0.34, 2.01
	Own (ref)	1.00	1.00, 1.00	1.00	1.00, 1.00	1.00	1.00, 1.00
**Annual household income**							
	<$20,000	2.12	1.04, 4.29	0.87	0.24, 3.15	0.87	0.13, 5.83
	$20,000 to <$35,000	1.82	0.99, 3.32	2.41	0.73, 7.95	1.31	0.33, 5.26
	$35,000 to <$50,000	1.56	0.82, 2.94	1.18	0.40, 3.55	1.02	0.28, 3.79
	$50,000 to <$75,000	1.12	0.59, 2.13	1.28	0.50, 3.28	2.22	1.04, 4.73
	>$75,000 (ref)	1.00	1.00, 1.00	1.00	1.00, 1.00	1.00	1.00, 1.00
**Age**							
	18-34	2.81	1.13, 7.00	3.91	0.59, 25.94	1.99	0.47, 8.45
	35-49	2.27	1.00, 5.17	1.65	0.27, 10.23	1.38	0.34, 5.65
	50-64	1.59	0.72, 3.49	1.70	0.25, 11.68	0.86	0.15, 4.97
	>65 (ref)	1.00	1.00, 1.00	1.00	1.00, 1.00	1.00	1.00, 1.00
**Sex**							
	Female	1.48	0.88, 2.49	4.31	1.78, 10.42	2.79	1.20, 6.51
	Male (ref)	1.00	1.00, 1.00	1.00	1.00, 1.00	1.00	1.00, 1.00
**Cancer experience**							
	Cancer diagnosis (self)	1.07	0.63, 1.81	2.93	1.00, 8.63	1.36	0.62, 2.99
	No cancer diagnosis (ref)	1.00	1.00, 1.00	1.00	1.00, 1.00	1.00	1.00, 1.00
	Family history of cancer	1.08	0.72, 1.62	1.14	0.52, 2.53	2.96	1.00, 3.83
	No family history (ref)	1.00	1.00, 1.00	1.00	1.00, 1.00	1.00	1.00, 1.00

^a^All estimates are weighted. All models control for occupational status, marital status, children, health information–seeking (ever sought health information from any source), regular access to a health care provider, insurance status, health status, personal cancer history, and family history of cancer.

^b^For the online support group model, high school degree and no high school degree were collapsed into 1 predictor variable to increase cell size for analysis.

## Discussion

Being younger and female has consistently been predictive of increased use of eHealth [5,21,25,47]. Younger generations who have grown up with technology have been labeled “digital natives” and they are more comfortable using technology for everyday needs, including management of their health care needs. In comparison, older generations, labeled “digital immigrants,” have had to learn and acquire the necessary skills needed to navigate the Internet and are generally less comfortable using technology [48]. Females also tend to have increased eHealth utilization due in part to their higher engagement in both health care-related online activities and increased use of general social media, such as SNS [3-5,49]. This could be because of their role as the health care liaison for their family members.

Our analysis identifies specific proxies of SES that are more reliably associated with eHealth use. Education was more consistently predictive of eHealth use across the health care and information-seeking domains, whereas household income was somewhat less predictive across items and domains. Although both education and income have been used to describe SES, in considering technology use and health communication, our analysis suggests that education may be a more salient proxy as compared to income.

Furthermore, this distinction between common proxies for SES may offer insight into the fundamental drivers of online use for health and subsequently assist in the development of more effective interventions and programs. Divides among those less educated indicate that issues of health literacy and eHealth literacy may be important factors. Research is emerging that advocates matching eHealth technology to the eHealth literacy (defined as “the ability to seek, find, understand, and appraise health information from electronic sources and apply knowledge gained to addressing or solving a health problem”) of the intended user [50-52]. Future analyses of online communication-based interventions should better investigate and address issues pertaining to eHealth literacy in an effort to reduce communication inequalities across low-SES groups.

It is also important to examine the direction of the relationship between education and engagement in eHealth tasks. Lower levels of education were associated with increased use of social media for health, whereas higher levels of education were associated with increased use of the Internet to engage in health care-related activities and search for information—much like the patterns we see for static Web 1.0 engagement. Similar patterns are seen in the Pew Internet & American Life Project’s 2013 Health Online study in that higher percentages of non-Hispanic blacks (35%) and Hispanics (38%) reported using their mobile phones to access health information compared to non-Hispanic whites (27%) [5]. These differences in use across domains may indicate potential inequalities in not only the quality of information obtained, but also patients’ engagement with the health care system. Further empirical examination is warranted to better objectively assess quality of health information shared via social media compared with more basic Web 1.0 sites as well as quality and satisfaction of care between those that engage in eHealth care and those that do not.

On the other hand, our study also indicates the potential for eHealth technologies to aid in reducing communication inequalities and disparities in health. Our data found no racial divides among the most vulnerable groups in eHealth use once access was achieved; there was only increased use of downloading information to a mobile device and looking online for a health care provider among other race individuals. This finding is consistent with several prior studies [5,25,35] and points to the opportunity to better explore the association between eHealth utilization and health outcomes among Hispanic and non-Hispanic blacks more directly.

Although our research is an important addition to the literature, we note its limitations. First, the low survey response rate may increase sampling error in our estimates; however, overall sampling coverage was enhanced through the stratified design. Also, because this was a cross-sectional survey study, it is challenging to account for unmeasured confounding variables. Future studies should examine potential factors related to those sociodemographic variables that were predictive of our outcomes, such as literacy, to determine their role in eHealth use. In addition, our study does not attempt to examine factors that may moderate or perhaps mediate the main associations presented within this analysis. For example, past research has examined the role of trust in terms of patients’ trust of the Internet as well as their own physicians and/or the health care system [53,54]. Examination of these psychographic variables is an important addition in better understanding the complex predictors of eHealth use. Yet, with such persistent inequalities in health, we cannot underestimate the importance of maintaining a constant understanding of eHealth use by sociodemographic characteristics.

A more qualitative examination into the rationale offered by patients for their eHealth utilization would build upon this work and offer a more robust understanding of why certain groups do and do not use the Internet for health purposes. A recent study conducted by the Pew Internet & American Life Project reports that 36% of non-Internet users cited that they did not use the Internet because they did not think that it was relevant to them, whereas 32% reported difficultly in use [55]. Investigation as to whether or not these reasons are applicable to eHealth use is warranted.

This study illustrates that lower SES, older, and male online US adults were less likely to engage in a number of eHealth activities compared to their counterparts. In an effort to reduce existing disparities in health outcomes, clinical and public health communication strategies should be attuned to these differences in online use.

Our results have important implications for clinical care and public health communication efforts attempting to leverage Web 2.0 and 3.0 platforms. It is evident that a one size/platform would not fit all, as significant demographic and individual factors influence eHealth engagement. For example, campaigns and interventions targeting women or younger populations may see success in utilizing user-generated content and sharing sites. Whereas, in the current Chronic Care Model of medical care, offline materials should perhaps supplement online health information if practitioners would like to ensure equal access to information across educational strata, age, and sex.

## Abbreviations

HINTS: Health Information National Trends Survey
SES: socioeconomic status
SNS: social networking sites
